# Mortality and Suicide Risk in Treatment-Resistant Depression: An Observational Study of the Long-Term Impact of Intervention

**DOI:** 10.1371/journal.pone.0048002

**Published:** 2012-10-25

**Authors:** Bryan Olin, Amara K. Jayewardene, Mark Bunker, Francisco Moreno

**Affiliations:** 1 Cyberonics, Inc, Houston, Texas, United States of America; 2 Department of Psychiatry, The University of Arizona, Tucson, Arizona, United States of America; Peking University, China

## Abstract

Major depressive disorder is a common global disease that causes a significant societal burden. Most interventional studies of depression provide a limited assessment of the interventions on mortality and suicide risks. This study utilizes data from an observational registry of patients with major depressive disorder to determine the impact of intervention (vagus nerve stimulation or standard pharmacological/non-pharmacological therapy) and a latent factor, patient trajectory toward response, on mortality, suicide and suicidal ideation. A total of 636 patients were available for an intent-to-treat analysis of all-cause mortality, suicide and suicidal ideation. Patients treated with vagus nerve stimulation in addition to standard therapies experienced lower, but not statistically significant, all-cause mortality (vagus nerve stimulation 4.93 per 1,000 person-years vs. 10.02 per 1,000 patient years for treatment as usual) and suicide rates (vagus nerve stimulation 0.88 per 1,000 person-years vs. 1.61 per 1,000 patient years for treatment as usual). Treatment with vagus nerve stimulation produced a statistically lower relative risk of suicidal ideation 0.80, 95% confidence interval (0.68,0.95). Further, patients that responded to either treatment saw a 51% reduction in relative risk of suicidal behavior; relative risk and 95% confidence interval of 0.49 (0.41,0.58). In summary, we find that treatment with adjunctive vagus nerve stimulation can potentially lower the risk of all-cause mortality, suicide and suicide attempts.

## Introduction

Major depressive disorder (MDD) is very common, affecting about 121 million people worldwide [1]. In the United States, the lifetime prevalence of MDD is approximately 16% and the 12-month prevalence is approximately 7% [2]. Treatment-resistant depression (TRD), an often more severe and/or more chronic subset of MDD, is characterized by failure to respond to multiple therapeutic interventions, including non-pharmacologic treatments [3]. The STAR*D trial [4], a NIMH funded, large scale prospective study of over 3000 outpatients with nonpsychotic MDD demonstrated that up to 35% of patients could be considered to have TRD.

Major depressive disorder, particularly the treatment-resistant form, is characterized by significant financial burden to the individual and society resulting from lost productivity of both the patient with TRD and any caregivers, as well as increased direct health care utilization, including utilization associated with suicide attempts and mortality [5], [6]. Worldwide, MDD is the leading cause of years lived with disability and is projected to be the second leading cause of disability adjusted life-years (DALY) by 2020, where DALY measures the burden to the individual and society [7]. In addition, it is projected that self-inflicted injuries will be the 10th leading cause of death in 2020. Major depressive disorder and other comorbid psychiatric conditions have been shown to result in an average of 27 years of potential life lost, a measure of the risk of premature death [8]. A study in the United Kingdom observed that the average decrease in life expectancy for patients with recurrent major depressive disorder is 7 years lost for females and almost 11 years lost for males [9].

Most research on therapeutic interventions for MDD addresses the effectiveness and adverse events (including suicidality) associated with those treatments, with limited focus on mortality. Several studies examined excess mortality associated with major depressive disorder [8]–[16]. However, the patients in these studies often have a less chronic and less severe form of depression than patients with TRD or were in studies undertaken prior to the availability of the current generation of anti-depressants and new trends in polypharmacy, such as the addition of atypical antipsychotics or simulant medications to the standard antidepressant medication regimen. In addition, they were generally long-term community-based, observational studies that did not evaluate the impact of a therapeutic intervention.

This report characterizes mortality and suicide risks associated with treatment-resistant depression from an observational study of comparing the performance of standard pharmacological and non-pharmacological therapies (TAU) to vagus nerve stimulation (VNS+TAU) therapy adjunctive to standard therapies.

VNS Therapy® consists of a small pulse generator surgically implanted in the chest that delivers intermittent stimulation (typically 30 seconds on, 5 minutes off) via an electrode partially wrapped around the left vagus nerve in the mid-cervical region; the electrical signals are in turn processed in the nucleus tractus solitarius and relayed to various regions of the brain [17]–[19].

VNS has demonstrated antidepressant activity in animal models of depression [20] and in epilepsy studies where improvement in patient moods was seen independent of its impact on seizure activity [21], [22].

VNS therapy was first approved for the adjunctive treatment of drug-resistant epilepsy in 1993 (Europe) and 1997 (US). It was subsequently approved in 2003 (Europe) and 2005 (US) for the adjunctive long-term treatment of chronic or recurrent depression for patients who are experiencing a major depressive episode and have not had an adequate response to four or more adequate antidepressant treatments. For both indications, there are specific age restrictions associated with the US FDA approvals. The effectiveness of VNS Therapy in depression has been previously reported in the literature [23]–[31].

VNS Therapy has also been considered as a possible adjunctive treatment for traumatic brain injury (TBI). This signal was first noted in patients with post-traumatic epilepsy who were non responsive to surgery [32]. Subsequently, animal models of TBI have led to the theory that VNS may play a role in TBI, potentially mediated by its effects on the immune response [33], [34]. VNS Therapy’s ability to modulate the inflammatory response in both animal models [34], [35] and humans [36]–[39] could lead to approved applications in other immunologically mediated disease states.

Beyond the effects of VNS in the central nervous system, the modality has also been shown to have significant peripheral effects. Recently, a pilot study has been completed evaluating the effects of VNS Therapy in patients with congestive heart failure (CHF) [40]. Positive results in this pilot study led to larger feasibility study in CHF being undertaken and recently completed [41]. The positive effect in CHF has led to the postulation that VNS Therapy may have a positive impact in patients suffering from ventricular arrhythmias [42].

## Methods

### Objectives

This research uses the results of an observational study to characterize the all-cause mortality rate, suicide rate and rate of suicidal ideation in patients with TRD. These rates are then compared for two interventions, standard treatment-as-usual (TAU) pharmacotherapy, where all available therapeutic interventions are allowed, including electro convulsive therapy (ECT) and psychotherapy, and VNS Therapy adjunctive to treatment-as-usual pharmacotherapy (VNS+TAU).

### Description of Procedures or Investigations Undertaken

The TRD Registry (NCT00320372) is an ongoing, post-market surveillance study required by FDA as a condition of approval of the treatment-resistant depression indication for VNS Therapy to evaluate long-term patient outcomes.

The study is an observational, open-label, longitudinal, multi-center (45 US centers) registry of 500 patients with TRD treated with VNS+TAU and 300 patients with TRD treated with TAU. Patients are followed for 60 months, until withdrawal from the study, death or study completion.

Data collected included patient demographics and medical (especially psychiatric) history, concomitant medications and medication history and measures of clinical effectiveness including the Clinical Global Impression Improvement (CGI-I) [43], Montgomery-Åsberg Depression Rating Scale (MADRS) [44] and the Quick Inventory of Depressive Symptomatology Self-Report (QIDS-SR) [45]. Safety measures assessed included mortality, suicidal ideation and side effects. Mortality, including suicide, is assessed through long-term follow-up. Suicidal ideation is measured using the Assessment of Suicidality (AOS) and MADRS Item 10 (Score ≥4. “*Probably better off dead. Suicidal thoughts are common, and suicide is considered as a possible solution, but without specific plans or intention.*”). To ensure consistency in ratings, a central ratings group is used to rate patients for both the MADRS and AOS measures. Side effects were assessed using the Frequency, Intensity, and Burden of Side Effects-Rating (FIBSER) questionnaire [46].

### Participants

Patients were eligible for inclusion in the TRD Registry if the following criteria were met:

- Patient diagnosed with a current major depressive episode according to DSM-IV-TR criteria.
- Patient has been in the current depressive episode for 2 years or longer, or has had at least 3 lifetime episodes including the current MDE.
- Patient has had an inadequate response to 4 or more adequate anti-depressive treatments.
- The patient has a Clinical Global Impression Severity of illness score (CGI-S) of moderately ill (score of 4) or greater.
- The patient must be able to provide informed consent and complete all forms.

Patients were excluded from the TRD Registry if they met one or more of the following criteria:

- Patient has a history of schizophrenia, schizoaffective disorder, any other psychotic disorder, or a current major depressive episode that includes psychotic features; or is currently psychotic.
- Patient is currently enrolled in a double blind investigational study
- Patient has previously received VNS therapy.
- Patient has a history of rapid cycling bipolar disorder.

### Ethics

The TRD Registry was approved by the Western Institutional Review Board (WIRB) as well as the following local Institutional Review Boards (IRB): Advocate HealthCare IRB (Park Ridge, IL), Baylor College of Medicine IRB (Houston, TX), Cedars-Sinai Institutional Review Board (CSMC IRB) (Beverly Hills, CA), Jamaica Hospital Medical Center IRB (Jamaica, NY), KU School of Medicine-Wichita Human Subjects Committee (Wichita, KS), Loma Linda University IRB (Loma Linda, CA), Medical College of Wisconsin Froedtert Hospital IRB (Milwaukee, WI), Medical University of South Carolina (Charleston, SC), New York State Psychiatric Institute IRB (New York, NY), NorthShore University Health System Research Institute IRB (Evanston, IL), Oregon Health and Science University Research Integrity Office (Portland, OR), Partners Human Research Committee (Boston, MA), SUNY Upstate IRB (Syracuse, NY), Sutter Health Central Area Institutional Review Committee (IRC) (Sacramento, CA), The University of Arizona IRB (Tucson, AZ), The University of Utah IRB Research Administration Building (Lake City, UT), University Hospitals Case Medical Center IRB (Cleveland, Ohio), University of Connecticut Health Center IRB (Farmington, CT), University of Massachusetts Medical School IRB (Worcester, MA), University of Mississippi Medical Center IRB (Jackson, MS), University of Pennsylvania IRB (Philadelphia, PA), University of Texas Health Science Center San Antonio IRB (San Antonio, TX), University of Texas Southwestern Medical Center IRB (Dallas, TX), Wake Forest University Health Sciences IRB (Winston-Salem, NC) and Washington University Human Research Protection Office (St. Louis, MO).

Informed consent was obtained from all enrolled patients.

### Statistical Methods

Patient demographics and baseline characteristics were summarized using descriptive statistics for the patients in each clinical study. Summary statistics include N, mean, standard deviation, median, and range (minimum, maximum) for continuous variables. Frequencies and percentages are used for summarizing categorical variables.

Absolute risk of mortality or suicide and rate of suicidal ideation were quantified as the number of events divided by the total exposure time to treatment. In addition, time spent in a state of non-response, clinical benefit (MADRS percentage decrease from baseline greater than 25% but less than 50%) and response (MADRS percentage decrease from baseline of 50% or more) was determined.

Due to the observational nature of these data, stratification was used to control for potential confounding factors.

A simple stratified analysis was conducted for mortality and suicide based on age group at the time of latest follow-up or death (under 40, 40–65 and 65 and older). Standardized mortality ratios (SMR), the observed number of deaths in the study population divided by the expected number of deaths in the US population, were calculated. The expected number of deaths was determined from published age-specific US mortality and suicide rates (Tables III, 3 and 10 of reference [47]). A SMR that exceeds 1 indicates that the study population has excess mortality relative to the US population.

A model for propensity scores [48] was identified using stepwise logistic regression methods on the binary outcome of treatment assignment, VNS+TAU treated = 1, TAU treated = 0. The significance level to enter (0.3 and 0.2) and significance level to remain (0.35 and 0.1) in the model were varied to assess the impact of these factors on model choice. The model considered the potential confounders in Table 1 and their two-factor interactions. Balance was assessed by examining the F-tests (continuous variables) or Cochran-Mantel-Haenszel (dichotomous or polytomous variables). The propensity scores were stratified into quintiles 1–5.

**Table 1:** Factors used for propensity score adjustment.

Age	Age at onset of depression
Age at first diagnosis of MDD	Ethnicity
Gender	Height
Weight	# depressive episodes in lifetime including current
# psychiatric hospitalizations in lifetime	duration of illness
length of current depressive episode	# lifetime suicide attempts
# suicide attempts in current episode	# failed treatment courses
Electroconvulsive therapy history (Y/N)	Primary diagnosis
Baseline CGI-S	Baseline MADRS
Baseline QIDS-SR	

A second stratified analysis of mortality and suicidal ideation was performed treating the propensity score quintiles as strata [48], thereby adjusting for baseline confounders. Finally, we stratified suicidal ideation by both propensity score quintiles and treatment response (non-response, clinical benefit and response).

For stratified analyses, crude rates and standardized rates were calculated using standard methods [49]. The treatment groups were standardized against overall exposure for the entire population of patients in the study. The Byar approximation for confidence intervals for rates and SMRs was used [49].

A more parsimonious approach was taken to estimate standardized rates and relative risks for suicidality, allowing the assessment of the impact of treatment and whether the patient’s outcome trajectory led to response or non-response. This involved fitting a marginal structural model [50] to the logarithm of the count of suicidal ideations with an offset of the logarithm of patient-years (converting the count to a rate) and explanatory variables of treatment (VNS+TAU or TAU), response (responder vs. non-responder) and the treatment by response interaction. Propensity scores were used as weights. The significance of the interaction was assessed and if non-significant was dropped from the final model. Confidence intervals for the relative rate ratios of suicidality of VNS+TAU compared to TAU and responders compared to non-responders are provided.

Two additional analyses were performed on medications and side effects. We examined patterns in changes in therapy over the course of the study to determine whether or not these differed between groups, which could indicate a potential bias. The FIBSER burden was tabulated longitudinally to determine if the side effect profile differed between the two groups. A score of 0–2 for burden of side effects is an acceptable side-effect burden usually requiring no treatment adjustment. A score of 3 or 4 indicates moderate side-effect burden that should be evaluated further and an adjustment such as a dose decrease considered. A score of 5 or 6 indicates a high burden warranting a change such as dose decrease, switching, or direct treatment of the side effect(s). [51]. Both analyses were qualitative in nature.

A significance level of 5% was used for all analyses. Statistical analysis was performed using SAS version 9.1.3 (SAS Institute, Cary, North Carolina) and Microsoft Excel.

## Results

### Patient Characteristics

**Figure 1:**
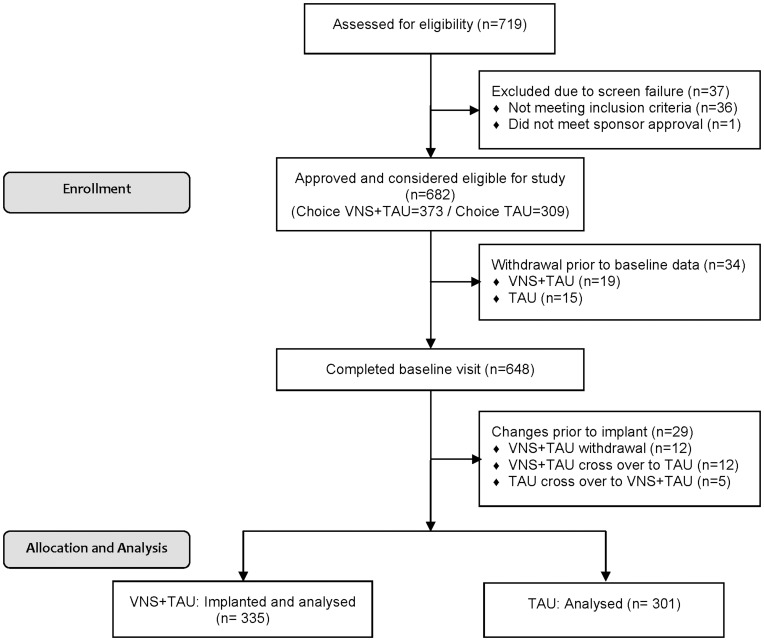
TRD Registry Patient Enrollment Flow Chart.

Figure 1 describes the patient flow during the course of the study. A total of 719 patients were assessed for eligibility; 682 were determined eligible and were enrolled in the study at 45 sites. After completing the screening visit, patients selected VNS+TAU (373) or TAU (309) options based upon which they believed was the best medical treatment. In May 2007, after the study had started, the Center for Medicaid Services issued a ‘non-coverage’ decision for VNS in TRD. This limitation in access and reimbursement caused treatment arm changes by precluding some patients from being implanted with VNS Therapy. A total of 34 patients exited the study prior to supplying any baseline data. After accounting for exits prior to implant and cross-overs, all 335 patients treated with VNS+TAU and 301 patients treated with TAU are considered in the subsequent intent-to-treat analyses. Patients in the VNS+TAU group have been followed for an average of 3.2 years vs. 2.1 years for the TAU group.

Table 2 summarizes the baseline characteristics of the study participants by treatment group.

**Table 2:** TRD Registry Baseline Demographics and Clinical Data.

	Overall	
Baseline Demographics	TAU^1^	VNS+TAU^2^
	(N = 301)	(N = 335)
Age (Yrs.)	49.8±11.1	48.8±10.4
% Female	70.1%	68.4%
% Caucasian	91.0%	96.4%
Age of Onset (Yrs.)	21.1±11.4	20.7±12.1
Age of First Diagnosis (Yrs.)	29.5±11.9	29.0±10.9
Length of Illness (Yrs.)	28.7±13.8	28.1±13.2
Length of Current Episode (Yrs.)	8.5±11.0	7.1±8.7
% ECT History	45.20%	58.20%
# Previous Drug Treatments	7.3±2.9	8.0±3.1
# Hospitalizations	1.9±4.7	2.8±4.6
# Lifetime Suicide Attempts	1.2±2.4	2.1±4.4
# Current Suicide Attempts	0.5±1.5	0.6±1.7
Primary Diagnosis of Current MDE		
Major Depressive Disorder	76.40%	71.10%
Bipolar Disorder	23.60%	28.90%
Baseline MADRS	29.4±6.9	33.1±7.9
Baseline CGI Severity	4.7±0.7	5.2±0.8
Baseline QIDS	15.6±4.9	18.3±4.7
Baseline % AOS^3^ Suicidal	1.30%	8.40%
Total Person-Years	646	1,114.40

1 All Treatment as Usual patients.

2 All VNS implanted patients.

3 Assessment of Suicidality (AOS).

The two treatment groups are well balanced for age, gender and length of illness. However, several baseline characteristics show notable differences. Patients treated with VNS+TAU have a more severe disease state than patients in the TAU group: increased history of ECT utilization (58% vs. 45%), a greater number of previous medications tried (8.0 vs. 7.3) and greater rate of psychiatric hospitalizations (2.8 vs. 1.9). We note that the greater use of prior treatment trials (ECT and medications) in the VNS+TAU likely reflects that the majority chose VNS+TAU as a final alternative when all other treatments had failed.

This more severe illness is corroborated with through baseline assessments of depressive illness as the MADRS and CGI-S scores for the VNS+TAU and TAU groups show clinically significant differences of 4 and 0.5 points, respectively. Further, the elevated rate of previous suicide attempts for the VNS+TAU group (2.1 vs. 1.2) is confirmed by the increased percentage of VNS+TAU patients who exhibit suicidal ideations at baseline as assessed by the Assessment of Suicidality *(“Has the patient made a suicidal gesture or attempt since the last visit?”* Yes or No) (8.5% vs. 1.5%).

To further assess the comparability of the two treatment groups, we examined medical history (Table S1) and family history of drug abuse and psychiatric illness (Table 3).

**Table 3:** TRD Registry Family History of Mental Illness.

		TAU	VNS+TAU
Psychiatric Issue	Family Relationship	(N = 301)	(N = 335)
Bipolar Disorder	First Degree Relatives	27.0%	23.0%
	Second Degree Relatives	17.3%	16.1%
Depression Disorder	First Degree Relatives	72.3%	72.6%
	Second Degree Relatives	51.1%	46.8%
Schizophrenia	First Degree Relatives	6.1%	4.9%
	Second Degree Relatives	9.4%	6.0%
Alcohol Abuse	First Degree Relatives	50.7%	44.7%
	Second Degree Relatives	50.0%	43.2%
Substance Abuse	First Degree Relatives	29.9%	22.5%
	Second Degree Relatives	21.6%	16.4%

The percentage of patients with histories of various illnesses was similar for the VNS+TAU and TAU groups. The exceptions were that the VNS+TAU group had a greater percentage of patients with current diagnoses of irritable bowel syndrome, hyperthyroidism and irregular heart rates at baseline. Both irritable bowel syndrome and hyperthyroidism are chronic health conditions that can be co-occurring with depression and can exacerbate symptoms and complicate a patient’s response to treatment. The higher baseline prevalence of irregular heart rates in the VNS+TAU treatment group may have increased the risk for cardiac death. This may have been mitigated by the potentially cardio-protective impact of vagus nerve stimulation noted earlier.

The TAU group had a greater percentage of patients with current diagnoses of cancer. One of the patients with a diagnosis of thyroid cancer did in fact die from liver cancer during the study. However, none of these issues would likely have impacted or biased measures of suicidality.

Similarly, we identified no significant trends in the family history of substance abuse and psychiatric illness that would indicate any differences between treatment groups.

The propensity score model resulted in a model utilizing terms for race (Caucasian vs. Other), length of current depressive episode, number of lifetime suicide attempts, ECT history (yes or no), baseline scores for the CGI-S, MADRS and QIDS-SR and two factor interactions between baseline CGI-S and ECT history and baseline QIDS-SR and the number of lifetime suicide attempts. None of the Cochran-Mantel-Haenszel or F-tests comparing treatments or treatment by propensity score quintile interaction were significant indicating that subclassification by propensity score quintiles was effective in removing bias.

Table 4 shows the number of patients per propensity score quintile along with the demographic characteristics. Basic demographic characteristics are similar. Note the marked gradation of disease state severity from propensity score quintile 1 to quintile 5 and that this composite measure confirms that the more severely ill patients were assigned to VNS+TAU.

**Table 4:** TRD Registry Baseline Demographics and Clinical Data by Propensity Score Quintile.

	Propensity Score Quintile^1^				
Baseline Demographics	1	2	3	4	5
	(N = 122; 22/100^2^)	(N = 122; 54/68)	(N = 122; 68/54)	(N = 122; 85/37)	(N = 122; 98/24)
Age (Yrs.)	50.3±11.8	49.4±11.23	51.5±10.2	47.6±9.5	47.8±10.1
% Female	74.6%	63.9%	65.6%	64.8%	74.6%
% Caucasian	82.8%	91.0%	99.2%	99.2%	97.5%
Age of Onset (Yrs.)	20.6±11.4	20.8±10.7	22.3±12.5	21.1±13.0	20.4±11.9
Age of First Diagnosis (Yrs.)	29.1±11.9	29.3±10.2	32.0±11.8	28.0±11.7	28.5±10.7
Length of Illness (Yrs.)	29.6±13.7	28.6±13.2	29.1±14.5	26.5±12.5	27.5±13.5
Length of Current Episode (Yrs.)	10.4±13.3	9.7±11.2	6.0±7.7	6.0±6.8	6.5±7.2
% ECT History	11.5%	62.3%	61.5%	71.3%	53.3%
# Previous Drug Treatments	6.7±2.4	7.6±3.3	8.2±2.8	8.3±3.3	7.8±3.0
# Hospitalizations	0.9±2.1	1.7±2.6	2.2±6.3	3.1±4.6	4.0±6.0
# Lifetime Suicide Attempts	1.0±2.4	1.0±1.9	0.8±1.7	1.6±4.3	3.9±5.3
# Current Suicide Attempts	0.5±2.1	0.2±0.6	0.2±0.7	0.5±0.9	1.3±2.6
Primary Diagnosis of Current MDE^3^					
Major Depressive Disorder	77.90%	78.70%	72.10%	69.70%	68.00%
Bipolar Disorder	22.10%	21.30%	27.90%	30.30%	32.00%
Baseline MADRS	26.7±6.8	28.1±7.7	30.8±5.4	33.7±6.5	37.6±6.8
Baseline CGI Severity	4.1±0.2	4.6±0.6	4.9±0.5	5.4±0.6	5.8±0.7
Baseline QIDS-SR	13.6±4.8	14.3±4.7	17.2±4.0	19.0±4.1	21.2±3.1
Baseline % AOS^4^ Suicidal	3.3%	3.3%	4.9%	7.4%	7.4%
Total Person-Years	224.70	274.70	266.20	305.30	292.80

1 26 patients had missing values and propensity scores could not be calculated.

2 Split between VNS and TAU patients, e.g., in quintile 1 there were 22 VNS+TAU and 100 TAU patients.

3 MDE (Mood Disorder Episode).

4 Assessment of Suicidality.

Significant increases across quintiles can be seen for hospitalizations, suicidality (prior attempts during lifetime and current episode, as well as the percentage of suicidal patients at baseline) and baseline assessments (CGI-S, QIDS-SR and MADRS). Note also that the percentage of patients with a bipolar diagnosis increases with propensity score quintile.

### Outcomes

Table 5 summarizes all-cause mortality and suicide rates in the two treatment groups, overall and by age; both crude and standardized rates are provided, along with an analysis by each group. The standardized all-cause mortality (4.46 vs. 8.06 per 1,000 person years) and suicide rates (0.88 vs. 1.61 per 1,000 person-years) for patients treated with VNS+TAU are about half that of the patients treated with TAU alone, but they are not statistically lower due to the low mortality rate in both groups. Similar results were noted when stratifying by propensity score quintiles.

**Table 5:** Analysis of all-cause mortality and suicide rates, stratified by age: VNS+TAU and TAU TRD Registry Populations.

	Rate per 1,000 Person-Years with 95% Confidence Intervals					Standardized Mortality Ratio
	Crude	Standardized	<40	40–64	65+	
***VNS+TAU Population***						
Total Person-Years	1,114.4		130.5	882.7	101.2	
All-Cause Mortality	4.49 (1.45,10.47)	4.46 (0.00,9.41)	7.66 (0.10,42.63)	4.53 (1.22,11.60)	0.00 (0.00,36.25)	0.53 (0.17,1.23)
Suicide	0.90 (0.01,4.99)	0.88 (0.00,3.05)	0.00 (0.00,28.11)	1.13 (0.01,6.30)	0.00 (0.00, 36.25)	5.72 (0.07,31.82)
***TAU Population***						
Total Person-Years	646.0		88.1	480.4	77.5	
All-Cause Mortality	7.74 (2.49,18.06)	8.06 (0.00,16.99)	0.00 (0.00,41.64)	10.41 (3.35,24.29)	0.00 (0.00,47.33)	0.81 (0.26,1.88)
Suicide	1.55 (0.02,8.61)	1.61 (0.00,5.61)	0.00 (0.00,41.64)	2.08 (0.03,11.58)	0.00 (0.00,47.33)	9.98 (0.13,55.55)

We note that most of the patient deaths occurred in the 40–65 age group. Overall excess mortality, as measured by the SMRs in Table 5, indicates that there appears to be no excess all-cause mortality relative to the US population; the 95% confidence intervals for the SMR contain one for both treatment groups; VNS+TAU: 0.53 (0.17,1.23) and TAU: 0.81 (0.26,1.88).

In contrast, as expected, both groups have a significantly elevated rate of suicide relative to the US population, approximately 6 times as great for the patients treated with VNS+TAU, 5.72 with 95% confidence interval (0.07,31.82), and 10 times as great for patients treated with TAU alone, 9.98 with 95% confidence interval (0.13,55.55). These results are similar to, if not slightly lower than, previous reports in similar populations of patients suffering from chronic depression.

Because the two treatment groups were well-matched on age, the more meaningful analysis is to stratify on the basis of propensity score quintiles to allow adjustment for other baseline confounders. Table 6 summarizes the stratified analysis of all-cause mortality and suicidal ideation as measured by both the Assessment of Suicidality and MADRS Item 10 score. A similar analysis of suicide was not meaningful due to the small number of events, one in each group. The results are similar to stratification by age, the VNS+TAU group had a mortality rate of 4.93 per 1,000 person-years, 95% confidence interval of 0.00 to 15.60, half of the rate for the TAU group, 10.02 per 1,000 patient years, with 95% confidence interval of 0.00 to 31.03. Further, we note that over 50% of the total exposure to treatment for VNS+TAU occurs for patients in propensity score quintiles 4 and 5, the most severely ill patients, in contrast to only 25% for TAU.

**Table 6:** Analysis of mortality and suicidal ideation event rates per 1,000 person-years, as measured by the Assessment of Suicidality (AOS) and MADRS Item 10, stratified by propensity quintile.

			Rate per 1,000 Person-Years with 95% Confidence Intervals				
			Crude			Standardized	
***VNS+TAU Population***							
Total Person-Years			1088.7^1^				
All-cause mortality			4.59 (1.48,10.72)			4.93 (0.00,15.60)	
Suicidal ideation (AOS)			64.30 (50.12,81.24)			61.25 (0.00,125.87)	
Suicidal ideation (MADRS Item 10)			256.27 (227.08,288.17)			234.27 (169.65,298.89)	
***TAU Population***							
Total Person-Years			627.2				
All-cause mortality			7.97 (2.57,18.60)			10.02 (0.00,31.03)	
Suicidal ideation (AOS)			47.83 (32.27,68.29)			47.28 (0.00,156.48)	
Suicidal ideation (MADRS Item 10)			204.08 (170.26,242.66)			261.78 (152.57,370.98)	
	**Rate per 1,000 Person-Years with 95% Confidence Intervals**						
	**1**	**2**		**3**	**4**		**5**
***VNS+TAU Population***							
Total Person-Years	79.6	177.2		227.8	297.0		307.1
All-cause mortality	12.56 (0.16,69.90)	0.00 (0.00,20.70)		0.00 (0.00,16.10)	10.10 (2.03,29.51)		3.26 (0.04,29.51)
Suicidal ideation (AOS)	62.81 (20.24,146.59)	50.79 (23.18,96.42)		21.95 (7.07,51.22)	43.77 (23.28,74.86)		123.74 (87.55,169.85)
Suicidal ideation (MADRS Item 10)	201.00 (114.82,326.44)	95.94 (55.85,153.61)		122.92 (81.66,177.65)	296.30 (237.63,365.05)		423.32 (353.67,502.66)
***TAU Population***							
Total Person-Years	186.5	167.4		116.6	88.4		68.3
All-cause mortality	5.36 (0.07,29.88)	0.00 (0.00,21.92)		17.15 (1.93,61.93)	11.31 (0.15,62.94)		14.64 (0.19,81.46)
Suicidal ideation (AOS)	64.34 (33.21,112.40)	17.92 (3.60,52.36)		60.03(24.05,123.70)	67.87 (24.78,147.74)		29.28 (3.29,105.72)
Suicidal ideation (MADRS Item 10)	128.69 (84.43,191.48)	95.58 (54.60,155.23)		197.26 (125.00,296.00)	429.86 (304.157,590.04)		395.322 (260.45,575.19)

1 Some patients had missing data at baseline leading to a missing propensity score.

Table 6 shows that suicidal ideation trends toward a higher rate at the more severe illness level. This is true with the MADRS Item 10 more so than for the Assessment of Suicidality, indicating that the MADRS Item 10 may be a more sensitive indicator of suicidal ideation and is better correlated with disease state. This is not surprising given that the likelihood of suicidal behavior was higher for patients in propensity quintiles 4 and 5, based on examination of the number of suicide attempts during the patients’ lifetime and current episode, lifetime as well as the assessment of suicidality.

Patients treated with VNS+TAU have a 10–20% reduction in the risk of suicidality as compared to patients treated with TAU alone for the MADRS Item 10, reaching significance with the marginal structural model (Table 7). In contrast, the Assessment of Suicidality is more variable and indicates that there is no statistically significant difference between treatment groups.

**Table 7:** Relative rate ratio comparing suicidal ideation rates for VNS+TAU to TAU.

Relative Rate Ratios	Standardized	Marginal Structural Model: Treatment Only	Marginal Structural Model: Treatment and MADRS Response
***VNS+TAU vs. TAU***			
AOS	1.30 (0.79,2.14)	1.44 (0.97,2.17)	1.47 (0.97,2.24)
MADRS Item 10	0.89 (0.54,1.48)	0.80 (0.68,0.95)	0.92 (0.77,1.09)
***Response vs. Non-response***			
AOS	0.38 (0.13,1.06)	NA	0.88 (0.58,1.33)
MADRS Item 10	0.01 (0.00,0.14)	NA	0.49 (0.41,0.58)

1 Not Calculated (NC) and Not Applicable (NA).

Table 7 shows the significant impact that response has on suicidal behavior. Response appears to be a stronger predictor of decreased suicidal behavior when included in the marginal structural model. As indicated in Table 7, patients who respond have a statistically significant 51% lower suicide risk than non-responders as measured by the MADRS Item 10; effects as measured by the standardized rates are even more pronounced. This is heavily weighted by the VNS+TAU group, which had the higher response rate, and a lower rate for decreased suicidal behavior, suicide and all-cause mortality. Even clinical benefit, having a 25–50% reduction in MADRS, appears to reduce suicidal behavior, as suggested by Table 8.

**Table 8:** Analysis of suicidal ideation rates per 1,000 person-years, as measured by the Assessment of Suicidality (AOS) and MADRS Item 10, stratified by treatment response and propensity score quintile.

	Rate per 1,000 Person-Years with 95% Confidence Intervals					
	No Benefit or Response		Clinical Benefit		Response	
	Crude	Standardized	Crude	Standardized	Crude	Standardized
***VNS+TAU Population***						
Total Person-Years	326.2		228.6		307.1	
AOS	88.90 (59.53,127.68)	76.02 (7.88,144.16)	100.61 (63.76,150.98)	68.07 (0.00,190.09)	29.31 (13.37,55.64)	26.59 (0.00,65.00)
MADRS Item 10	705.09 (616.90,802.35)	633.72 (426.50,840.94)	205.60 (151.05,273.41)	181.19 (64.02,298.37)	6.51 (0.73,23.51)	5.54 (0.00,22.42)
***TAU Population***						
Total Person-Years	291.5		120		90.3	
AOS	58.32 (33.95,93.38)	57.01 (0.00,124.57)	50.00 (18.26,108.83)	68.07 (0.00,190.09)	33.22 (6.68,97.07)	26.51 (0.00,97.40)
MADRS Item 10	397.94 (328.82,477.30)	529.70 (296.43,762.96)	100.00 (51.61,174.69)	131.99 (0.00,305.52)	0.00 (0.00,40.62)	0.00 NC^1^

1 NC: Not calculated both point estimates were 0, leading to a 0 standard error.

Given that TAU allowed for the use of any available therapy, we report in Table 9 the median, minimum and maximum number of times therapies were added, stopped or had dosage increases or decreases throughout the study per patient, for each treatment group. Examination of this table reveals that the profiles of therapeutic interventions were similar for both treatment groups, so opportunities for bias would be limited at best. Interestingly, we note that more patients in the VNS+TAU group were able to stop taking therapies than in the TAU group. Further study of this phenomenon is warranted.

**Table 9:** Tabulation of Changes in Therapy.

Therapy	Number of Patients		Additions^1^		Stops		Decreased Dose		Increased Dose	
	TAU	VNS+TAU	TAU	VNS+TAU	TAU	VNS+TAU	TAU	VNS+TAU	TAU	VNS+TAU
Antidepressant	283	314	0 (0 to 4)	0 (0 to 5)	0 (0 to 4)	1 (0 to 5)	0 (0 to 2)	0 (0 to 2)	0 (0 to 3)	0 (0 to 3)
Antiepileptic	137	205	0 (0 to 2)	0 (0 to 2)	0 (0 to 2)	1 (0 to 3)	0 (0 to 4)	0 (0 to 3)	0 (0 to 2)	0 (0 to 3)
Antipsychotic	4	13	None	0 (0 to 1)	0.5 (0 to 1)	1 (0 to 2)	None	0 (0 to 1)	None	None
Anxiolytic	121	164	0 (0 to 2)	0 (0 to 1)	0 (0 to 2)	0 (0 to 2)	0 (0 to 1)	0 (0 to 2)	0 (0 to 1)	0 (0 to 2)
Atypical Antipsychotic	150	194	0 (0 to 2)	0 (0 to 2)	1 (0 to 2)	1 (0 to 3)	0 (0 to 1)	0 (0 to 2)	0 (0 to 3)	0 (0 to 3)
ECT	24	20	0 (0 to 1)	0 (0 to 2)	1 (0 to 1)	1 (0 to 2)	NA	NA	NA	NA
Hypnotic	126	146	0 (0 to 2)	0 (0 to 4)	0 (0 to 2)	1 (0 to 4)	0 (0 to 1)	0 (0 to 1)	0 (0 to 1)	0 (0 to 2)
Lithium	40	49	0 (0 to 2)	0 (0 to 1)	0 (0 to 2)	1 (0 to 1)	0 (0 to 1)	0 (0 to 1)	0 (0 to 1)	0 (0 to 2)
Psychostimulant	56	83	0 (0 to 1)	0 (0 to 3)	0 (0 to 3)	1 (0 to 3)	0 (0 to 1)	0 (0 to 2)	0 (0 to 1)	0 (0 to 2)
Therapy	13	15	1 (0 to 2)	0 (0 to 1)	1 (0 to 2)	1 (0 to 2)	NA	NA	NA	NA
Thyroid Suppl.	39	65	0 (0 to 1)	0 (0 to 2)	0 (0 to 1)	0 (0 to 1)	0 (0 to 1)	0 (0 to 1)	0 (0 to 1)	0 (0 to 1)

1 The median, minimum and maximum number of therapeutic additions are provided; similarly for the analysis of therapies stopped, decreased or increased.

Similarly, an examination of the side effect profiles as measured by the FIBSER (Table 10), shows that the percentage of unacceptable site effects for VNS+TAU is higher than TAU, but that this difference dissipates over time. This is consistent with the experience in drug-resistant epilepsy and current product labeling [52], [53].

**Table 10:** TRD Registry Analysis of Frequency, Intensity and Burden of Side Effects Rating (FIBSER).

Visit(Month)	TAU			VNS+TAU		
	Acceptable (0–2)	Moderate (3–4)	Unacceptable (5–6)	Acceptable (0–2)	Moderate (3–4)	Unacceptable (5–6)
0	76.5%	19.4%	4.1%	64.3%	29.9%	5.7%
3	79.3%	18.0%	3.6%	73.4%	21.5%	5.1%
6	76.8%	20.6%	2.5%	79.5%	15.6%	4.7%
9	71.4%	14.0%	4.7%	76.8%	19.2%	4.1%
12	76.7%	18.0%	5.4%	74.6%	21.6%	3.9%
18	86.2%	10.2%	3.6%	74.1%	22.4%	3.5%
24	75.4%	20.1%	4.4%	76.8%	21.0%	2.1%
36	86.0%	10.0%	4.0%	81.4%	14.4%	4.2%

## Discussion

Key findings of our study were that patients treated with VNS+TAU experienced lower suicide risk and a potential signal toward decreased all-cause mortality rate. Additionally, the suicide and all-cause mortality rates in our study are generally consistent, or lower than, those reported in other large longitudinal studies of depression patients previously cited. Notably, the suicide rate for VNS+TAU is about half the standardized mortality rate observed in the TAU alone group. This lowered all-cause mortality rate is consistent with a recent report comparing patients with TRD in the US Medicare system treated with TAU (46.2 per 1,000 person-years) with patients treated with VNS+TAU (19.9 per 1,000 person-years) [54].

These data suggest that both response to (≥50% reduction in MADRS score) and partial clinical benefit (25 to 49% reduction in MADRS score) from VNS+TAU reduce the risk of suicidal behavior. Table 8 shows that the reduction in incidence of suicidal behavior is not restricted to responders but does occur for a continuum of antidepressant response for patients who show a partial clinical benefit. Most existing studies of depression therapies focus on response and remission of depressive symptoms as the primary clinical endpoints. This continues to be the appropriate gold standard; however, our study indicates that, in this treatment-resistant population, obtaining response or even moderate reduction of depression symptoms can effectively mitigate suicidal behavior.

These results were obtained in the backdrop of a unique study that includes a large number of patients with severe and chronic MDD who were followed for two to three years for two interventions. In contrast, most published data assessing interventions, generally pharmacotherapy, involve much shorter follow-up periods, usually one to two months and do not include TRD patients [16]. Studies with longer follow-up periods are observational cohort studies that aim to describe mortality and suicide rates and assess the impact of the disease state severity or demographic factors. The present study allows both objectives to be met: longer follow-up and assessment of the impact of a successful or unsuccessful therapeutic intervention has on mortality and suicide risk.

In addition, based on baseline CGI-S, QIDS-SR and MADRS scores, the patients in this study are more chronically and often more severely ill than many other patients studied in other papers previously referenced. For example, the average CGI-S across 207 anti-depressant trials is 4.1 to 4.6, as compared to the averages of 4.7 (TAU) and 5.2 (VNS+TAU) in our study [16]. Despite this difference in severity of illness, the suicide rate is less than that reported in most large series of monotherapy RCTs for the various medications used in patients treated in the TAU group. In addition, all available therapeutic interventions were allowed, including electro convulsive therapy (ECT) and psychotherapy, in the TAU group for this study.

The greater effect in treatment responders suggests that adjunctive VNS therapy may act synergistically with pharmacotherapy to improve depressive symptoms and decrease suicidal ideation, ultimately decreasing the incidence of suicide and premature death in patients with TRD. This is significant as the only other therapy associated with a similar reduction in suicidal ideation and suicide risk in patients with MDD, as well as bipolar disorder, is lithium as suggested by placebo-controlled trials [55] and a number of reviews [56], [57].

Severely ill patients (5^th^ propensity score quintile) also see a significant benefit from adjunctive VNS therapy, e.g., reduction in suicide risk. However, this reduction is not to the same degree as less severely ill patients, indicating that patients with a greater degree of illness severity may need an appropriate increase in frequency of physician follow up initially. This continued level of suicide risk is not surprising given that this group had twice the rate of suicide attempts and hospitalizations at baseline.

With respect to mortality rates, fortunately, only a small number of participants died during the study; details are provided in Table S2. This, combined with the variety of causes with several non-natural causes (homicide, suicide, accident) and several not known, precludes us from assessing if adjunctive VNS therapy leads to a decrease in mortality by natural causes potentially influenced by the vagal system. This limitation also prevented an assessment of whether mortality is dependent upon therapeutic response.

Although this is a limitation of the study at this time, it does provide an interesting potential signal to be explored as additional data are collected. The mechanism of action for such a potential may be solely related to differential antidepressant response between groups. Alternatively, it may indicate that adjunctive VNS therapy has particular clinical utility for patients with TRD who are at increased risk of mortality resulting from conditions modulated by the vagus nerve, such as patients with cardiac and vascular insufficiencies or immunologically compromised patients.

Given the current awareness of increases in all-cause mortality in patients with severe mental illness, the current study addresses an important outcome variable often ignored in prospective clinical trials and prospective observations. The findings of our study are thus significant both from the prospective of clinical outcome as well as the public health burden imposed on society by these patients.

### Limitations

The key limitations of this study are that it is non-randomized and observational in nature. In particular, selection bias was present as patients were allowed to choose their allocation to VNS+TAU or TAU which created the difference in disease severity between the two treatment groups; more severely ill patients were more likely to desire VNS+TAU as they had exhausted most other treatment options.

The impact of this selection bias was assessed via stratification and would have theoretically increased the likelihood of non-response, suicidal ideation, suicide and mortality in the VNS+TAU group due to the allocation of more severely ill patients.

## Supporting Information

Table S1 **TRD Registry Medical History Data.**
(DOCX)

Table S2 **Baseline clinical detail on patient deaths.**
(DOCX)

## References

[1] World Health Organization (2011) Depression. Available: http://www.who.int/mental_health/management/depression/definition/en/, Accessed: 2011 January 25.

[2] Kessler RC, Berglund P, Demler O, Jin R, Koretz D, et al (2003) The epidemiology of major depressive disorder: results from the National Comorbidity Survey Replication (NCS-R). JAMA 289: 3095–3105. 12813115 10.1001/jama.289.23.3095

[3] AHRQ (2011) Nonpharmacologic Interventions for Treatment-Resistant Depression in Adults. Comparative Effectiveness Review Number 33. 22624165

[4] Rush AJ, Trivedi MH, Wisniewski SR, Nierenberg AA, Stewart JW, et al (2006) Acute and longer-term outcomes in depressed outpatients requiring one or several treatment steps: a STAR*D report. Am J Psychiatry 163: 1905–17. 17074942 10.1176/ajp.2006.163.11.1905

[5] Greden JF (2001) The Burden of Disease for Treatment-Resistant Depression. J Clin Psychiatry 62[Suppl 16]: 26–31. 11480881

[6] Culpepper L (2011) Understanding the burden of depression. J Clin Psychiatry 72(6): e19. 21733472 10.4088/JCP.10126tx1c

[7] Murray CJL, Lopez AD (1997) Alternative projections of mortality and disability by cause 1990–2020: Global Burden of Disease Study. The Lancet 349: 1498–1504. 10.1016/S0140-6736(96)07492-2 9167458

[8] Colton CW, Manderscheid RW (2006) Congruencies in increased mortality rates, years of potential life lost, and causes of death among public mental health clients in eight states. Prev Chronic Dis. Available: http://www.cdc.gov/pcd/issues/2006/apr/05_0180.htm Accessed: 2012 March 23. PMC1563985 16539783

[9] Chang C-K, Hayes RD, Perera G, Broadbent MTM, Fernandes AC, et al (2011) Life Expectancy at Birth for People with Serious Mental Illness and Other Major Disorders from a Secondary Mental Health Care Case Register in London. PLoS ONE 6(5): e19590 doi:10.1371/journal.pone.0019590. 21611123 10.1371/journal.pone.0019590 PMC3097201

[10] Berglund M, Nilsson K (1987) Mortality in severe depression: A prospective study including 103 suicides. Acta Psychiatr Scand 76: 372–38. 3425363 10.1111/j.1600-0447.1987.tb05621.x

[11] O’Leary DA, Lee AS (1996) Seven year prognosis in depression: mortality and readmission risk in the Nottingham ECT cohort. Br. J. Psychiatry 169: 423–429. 10.1192/bjp.169.4.423 8894191

[12] Harris EC, Barraclough B (1997) Suicide as an outcome for mental disorders. Br J Psychiatry 170: 205–228. 9229027 10.1192/bjp.170.3.205

[13] Harris EC, Barraclough B (1998) Excess mortality of mental disorder. Br J Psychiatry 173: 11–53. 9850203 10.1192/bjp.173.1.11

[14] Shergill SS, Robertson MM, Stein G, Bernadt M, Katona CLE (1999) Outcome in refractory depression. J. Affect Disord 54: 287–294. 10467973 10.1016/s0165-0327(98)00201-8

[15] Brâdvik L, Berglund M (2001) Late mortality in severe depression. Acta Psychiatr Scand 103: 111–116. 11167313 10.1034/j.1600-0447.2001.00212.x

[16] Hammad TA, Laughren TP, Racoosin JA (2006) Suicide Rates in Short-term Randomized Controlled Trials of Newer Antidepressants. J Clin Psychopharmacol 26 2: 203–207. 10.1097/01.jcp.0000203198.11453.95 16633153

[17] Henry TR (2002) Therapeutic mechanisms of vagus nerve stimulation. Neurology. 59: S3–14. 10.1212/wnl.59.6_suppl_4.s3 12270962

[18] George MS, Sackeim HA, Marangell LB, Husain MM, Z. Nahas Z, et al (2000) Vagus nerve stimulation. A potential therapy for resistant depression? Psychiatr. Clin. North Am. 23: 757–783. 10.1016/s0193-953x(05)70196-9 11147246

[19] Nemeroff CB, Mayberg HS, Krahl SE, McNamara J, Frazer A, et al (2006) VNS therapy in treatment-resistant depression: clinical evidence and putative neurobiological mechanisms. Neuropsychopharmacology 31: 1345–1355. 16641939 10.1038/sj.npp.1301082

[20] Krahl SE, Senanayake SS, Pekary AE, Sattin A (2004) Vagus nerve stimulation (VNS) is effective in a rat model of antidepressant action. J Psychiatr Res 38: 237–240. 15003428 10.1016/j.jpsychires.2003.11.005

[21] Harden CL, Pulver MC, Ravdin LD, Nikolov B, Halper JP, Labar DR (2000) A pilot study of mood in epilepsy patients treated with vagus nerve stimulation. Epilepsy Behav 1: 93–99. 12609137 10.1006/ebeh.2000.0046

[22] Elger G, Hoppe C, Falkai P, Rush AJ, Elger CE (2000) Vagus nerve stimulation is associated with mood improvements in epilepsy patients. Epilepsy Res. 42: 203–210. 10.1016/s0920-1211(00)00181-9 11074193

[23] Marangell LB, Rush AJ, George MS, Sackeim HA, Johnson CR, et al (2002) Vagus nerve stimulation (VNS) for major depressive episodes: one year outcomes. Biol Psychiatry 51: 280–287. 11958778 10.1016/s0006-3223(01)01343-9

[24] George MS, Rush AJ, Marangell LB, Sackeim HA, Brannan SK, et al (2005) A one-year comparison of vagus nerve stimulation with treatment as usual for treatment-resistant depression. Biol Psychiatry 58: 364–373. 16139582 10.1016/j.biopsych.2005.07.028

[25] Nahas Z, Marangell LB, Husain MM, Rush AJ, Sackeim HA, et al (2005) Two-year outcome of vagus nerve stimulation (VNS) for treatment of major depression episodes. J Clin Psychiatry 66: 1097–1104. 16187765 10.4088/jcp.v66n0902

[26] Rush AJ, Marangell LB, Sackeim HA, George MS, Brannan SK, et al (2005) Vagus nerve stimulation for treatment-resistant depression: a randomized, controlled acute phase trial. Biol Psychiatry 58: 347–354. 16139580 10.1016/j.biopsych.2005.05.025

[27] Rush AJ, Sackeim HA, Marangell LB, George MS, Brannan SK, et al (2005) Effects of 12 months of vagus nerve stimulation in treatment-resistant depression: a naturalistic study. Biol Psychiatry 58: 355–363. 16139581 10.1016/j.biopsych.2005.05.024

[28] Sackeim HA, Brannan SK, Rush AJ, George MS, Marangell LB, et al (2007) Durability of antidepressant response to vagus nerve stimulation (VNS™). Int J Neuropsychopharmacol 10: 817–826. 17288644 10.1017/S1461145706007425

[29] Bajbouj M, Merkl A, Schlaepfer TE, Frick C, Zobel A, et al (2010) Two-year outcome of vagus nerve stimulation in treatment-resistant depression. Journal of Clinical Psychopharmacology 30(3): 273–281. 20473062 10.1097/JCP.0b013e3181db8831

[30] Martin JLR, Martin-Sanchez E (2012) Systematic review and meta-analysis of vagus nerve stimulation in the treatment of depression: variable results based on study designs. European Psychiatry 27: 147–155. 22137776 10.1016/j.eurpsy.2011.07.006

[31] Daban C, Martinez-Aran A, Cruz N, Vieta E (2008) Safety and efficacy of Vagus Nerve Stimulation in treatment-resistant depression. A systematic review. J Affect Disord 110(1–2): 1–15. 18374988 10.1016/j.jad.2008.02.012

[32] Lee HO, Koh EJ, Oh YM, Park SS, Kwon KH, et al (2008) Effect of vagus nerve stimulation in post-traumatic epilepsy and failed epilepsy surgery: preliminary report. J Korean Neurosurg Soc 44: 196–198. 19096676 10.3340/jkns.2008.44.4.196 PMC2588308

[33] Kumaria A, Tolias CM (2012) Is there a role for vagus nerve stimulation therapy as a treatment of traumatic brain injury? British Journal of Neurosurgery 26(3): 316–320. 22404761 10.3109/02688697.2012.663517

[34] Lopez NE, Krzyzaniak M, Costantini TW, De Maio A, Baird A, et al (2012) Vagal nerve stimulation blocks peritoneal macrophage inflammatory responsiveness after severe burn injury. J Trauma Acute Care Surg 72(6): 1562–1566.22695423

[35] Mihaylova S, Killian A, Mayer K, Pullamsetti SS, Schermuly R, et al.. (2012) Effects of anti-inflammatory vagus nerve stimulation on the cerebral microcirculation in endotoxinemic rats. J Neuroinflammation 25;9(1): 183. [Epub ahead of print]. 10.1186/1742-2094-9-183 PMC3425315 22830560

[36] Corcoran C, Connor TJ, O’Keane V, Garland MR (2005) The effects of vagus nerve stimulation on pro- and anti-inflammatory cytokines in humans: a preliminary report. Neuroimmunomodulation 12(5): 307–9. 16166810 10.1159/000087109

[37] Majoie HJM, Rijkers K, Berfelo MW, Hulsman JARJ, Myint A, et al (2011) Vagus Nerve Stimulation in Refractory Epilepsy: Effects on Pro- and Anti-Inflammatory Cytokines in Peripheral Blood. Neuroimmunomodulation 18: 52–56. 20639683 10.1159/000315530

[38] Vagus Nerve Stimulation a New Approach in the Treatment of Crohn’s Disease (VNS), ClinicalTrials.gov Identifier: NCT01569503, Available: http://clinicaltrials.gov/ct2/show/NCT01569503, Accessed: 2012 August 29.

[39] Safety and Efficacy Vagal Nerve Stimulation in Patients With Rheumatoid Arthritis, ClinicalTrials.gov Identifier: NCT01552941, Available: http://clinicaltrials.gov/ct2/show/NCT01552941, Accessed: 2012 August 29.

[40] Schwartz PJ, De Ferraria GM, Sanzo A, Landolinaa M, Rordorfa R, et al (2008) Long term vagal stimulation in patients with advanced heart failure First experience in man. Eur J Heart Fail 10(9): 884–891. 18760668 10.1016/j.ejheart.2008.07.016

[41] De Ferrari GM, Crijns HJ, Borggrefe M, Milasinovic G, Smid J, et al (2011) CardioFit Multicenter Trial Investigators. Chronic vagus nerve stimulation: a new and promising therapeutic approach for chronic heart failure. Eur Heart J 32(7): 847–55. 21030409 10.1093/eurheartj/ehq391

[42] Brack KE, Winter J, Ng GA (2012) Mechanisms underlying the autonomic modulation of ventricular fibrillation initiation-tentative prophylactic properties of vagus nerve stimulation on malignant arrhythmias in heart failure. Heart Fail Rev. doi:10.1007/s10741-012-9314-2. 10.1007/s10741-012-9314-2 PMC3677978 22678767

[43] Guy W (1976) ECDEU Assessment Manual for Psychopharmacology. Rockville, MD: US Department of Health, Education, and Welfare.

[44] Montgomery SA, Asberg M (1979) A new depression scale designed to be sensitive to change. Br J Psychiatry 134: 382–9. 444788 10.1192/bjp.134.4.382

[45] Rush AJ, Trivedi MH, Ibrahim HM, Carmody TJ, Arnow B, et al (2003) The 16-item Quick Inventory of Depressive Symptomatology (QIDS) Clinician Rating (QIDS-C) and Self-Report (QIDS-SR): A psychometric evaluation in patients with chronic major depression. Biol Psychiatry 54: 573–583. 12946886 10.1016/s0006-3223(02)01866-8

[46] Wisniewski SR, Rush AJ, Balasubramani GK, Trivedi MH, Nierenberg AA (2006) Self-rated global measure of the frequency, intensity, and burden of side effects. J Psychiatr Pract 12: 71–79. 16728903 10.1097/00131746-200603000-00002

[47] Xu JQ, Kochanek KD, Murphy SL, Tejada-Vera B (2010) Deaths: Final data for 2007. National vital statistics reports; vol 58 no 19. Hyattsville, MD: National Center for Health Statistics. 25075874

[48] Rosenbaum PR, Rubin DB (1984) Reducing Bias in Observational Studies Using Subclassification on the Propensity Score. J Amer Stat Assoc 79: 516–524.

[49] Rothman KJ, Greenland S and Lash TL (2008) Modern Epidemiology, 3^rd^ edition. Oxford: Lippincott, Williams and Wilkins.

[50] Sato T, Matsuyama Y (2003) Marginal Structural Models as a Tool for Standardization. Epidemiology 14(6): 680–686. 14569183 10.1097/01.EDE.0000081989.82616.7d

[51] Trivedi MH (2007) Tools and strategies for ongoing assessment of depression: a measurement-based approach to remission. J Clin Psychiatry 70 Suppl 626–31. 10.4088/JCP.8133su1c.04 19922741

[52] Morris GL 3rd, Mueller WM (1999) Long-term treatment with vagus nerve stimulation in patients with refractory epilepsy. The Vagus Nerve Stimulation Study Group E01–E05. Neurology. 53(8): 1731–5. 10.1212/wnl.53.8.1731 10563620

[53] Part III - Depression Information VNS Therapy™ Pulse Generators. December 2008. Available: http://dynamic.cyberonics.com/manuals/, Accessed: 2012 August 29.

[54] Feldman RL, Dunner DL, Muller JS, Stone DA (2012) Medicare Patient Experience with Vagus Nerve Stimulation for Treatment Resistant Depression. J Med Econ doi:10.3111/13696998.2012.724745. 10.3111/13696998.2012.724745 22954061

[55] Lauterbach E, Felber W, Müller-Oerlinghausen B, Ahrens B, Bronisch T, et al (2008) Adjunctive lithium treatment in the prevention of suicidal behaviour in depressive disorders: a randomised, placebo-controlled, 1-year trial. Acta Psychiatr Scand 118(6): 469–79. 18808400 10.1111/j.1600-0447.2008.01266.x

[56] Baldessarini RJ, Tondo L, Davis P, Pompili M, Goodwin FK, et al (2006) Decreased risk of suicides and attempts during long-term lithium treatment: a meta-analytic review. Bipolar Disord 8(5 Pt 2): 625–39. 10.1111/j.1399-5618.2006.00344.x 17042835

[57] Guzzetta F, Tondo L, Centorrino F, Baldessarini RJ (2007) Lithium treatment reduces suicide risk in recurrent major depressive disorder. J Clin Psychiatry 68(3): 380–3. 17388706 10.4088/jcp.v68n0304

