# Supplementary material for: Mortality and Suicide Risk in Treatment-Resistant Depression: An Observational Study of the Long-Term Impact of Intervention
Source: PLoS One. 2012 Oct 25;7(10):e48002. doi: 10.1371/journal.pone.0048002 (PMC3485051; doi:10.1371/journal.pone.0048002)
Supplement: Table S1 — TRD Registry Medical History Data. (DOCX) [file pone.0048002.s001.docx]

|  |  | **TAU** | **VNS+TAU** |
| --- | --- | --- | --- |
| **Medical Illness** | **Status** | **N=301** | **N=335** |
| Diabetes | History | 2.7% | 2.4% |
|  | Current | 13.6% | 10.7% |
| Cancer | History | 9.6% | 6.9% |
|  | Current | 1.3% | 0.0% |
| Chronic Pain | History | 8.3% | 6.9% |
|  | Current | 36.2% | 36.1% |
| Osteoporosis | History | 1.0% | 0.6% |
|  | Current | 6.3% | 8.1% |
| Multiple Sclerosis | History | 0.0% | 0.0% |
|  | Current | 0.0% | 0.9% |
| Parkinson Disease | History | 0.3% | 0.0% |
|  | Current | 0.0% | 0.3% |
| Epilepsy | History | 0.7% | 0.6% |
|  | Current | 1.0% | 1.5% |
| Other Neuro Disorder | History | 3.0% | 2.7% |
|  | Current | 13.3% | 17.0% |
| Ulcers | History | 12.3% | 11.0% |
|  | Current | 2.0% | 3.3% |
| Irritable bowel Syndrome | History | 11.6% | 9.9% |
|  | Current | 14.0% | 19.1% |
| Other GI Disorder | History | 8.3% | 10.1% |
|  | Current | 24.6% | 29.0% |
| HIV AIDS | History | 0.0% | 0.3% |
|  | Current | 1.0% | 0.3% |
| Hypothyrodism | History | 6.0% | 2.7% |
|  | Current | 17.6% | 25.1% |
| Obstructive Lung Disorder | History | 0.7% | 0.9% |
|  | Current | 3.3% | 2.4% |
| Other Pulmonary Disorder | History | 3.3% | 5.4% |
|  | Current | 10.3% | 12.5% |
| Myocardial Infarction | History | 1.7% | 2.7% |
|  | Current | 0.0% | 0.0% |
| Hypertension | History | 5.0% | 2.1% |
|  | Current | 29.2% | 29.6% |
| Heart Rate Irregular | History | 7.6% | 4.2% |
|  | Current | 6.0% | 9.6% |
| Syncope | History | 8.6% | 4.8% |
|  | Current | 2.0% | 2.7% |
| Stroke | History | 3.0% | 3.0% |
|  | Current | 0.0% | 0.3% |
| Other Cardio Disorder | History | 0.0% | 0.0% |
|  | Current | 0.3% | 0.6% |
