# Supplementary material for: Mortality and Suicide Risk in Treatment-Resistant Depression: An Observational Study of the Long-Term Impact of Intervention
Source: PLoS One. 2012 Oct 25;7(10):e48002. doi: 10.1371/journal.pone.0048002 (PMC3485051; doi:10.1371/journal.pone.0048002)
Supplement: Table S2 — Baseline clinical detail on patient deaths. (DOCX) [file pone.0048002.s002.docx]

| **Treatment** | **Cause of Death** | **Age** | **Gender** | **Duration of Illness** | **Length of Current Episode** | **Prior ECT Use?** | **# of Mood Medications** | **# Hospitalizations** | **# Lifetime Suicide Attempts** | **CGI** | **MADRS** | **MADRS Item 10** | **AOS** |
| --- | --- | --- | --- | --- | --- | --- | --- | --- | --- | --- | --- | --- | --- |
| TAU | Myocardial Infarction | 58.2 | F | 47 | 7.5 | No | 6 | 0 | 1 | 5 | 26 | 3 | 1 |
| TAU | Myocardial Infarction | 52.4 | M | 39 | 0.3 | Yes | 7 | 2 | 4 | 5 | 42 | 2 | 0 |
| TAU | Unknown | 46.7 | M | 36 | 0.5 | No | 4 | 1 | 2 | 6 | 33 | 4 | 0 |
| TAU | Suicide | 47.6 | F | 27 | 3.8 | No | 11 | 2 | 1 | 5 | 28 | 1 | 0 |
| TAU | Liver cancer | 50.6 | F | 12 | 8.7 | No | 5 | 0 | 0 | 4 | 44 | 3 | 0 |
| VNS+TAU | Diabetes | 55.1 | F | 32 | 2.7 | No | 8 | 1 | 0 | 5 | 45 | 4 | 0 |
| VNS+TAU | Inconclusive autopsy report | 40.2 | F | 25 | 0.3 | Yes | 8 | 10 | 6 | 6 | 27 | 4 | 0 |
| VNS+TAU | Suicide | 51.7 | F | 24 | 0.4 | Yes | 4 | 4 | 1 | 4 | 25 | 5 | 0 |
| VNS+TAU | Accidental | 55.8 | F | 9 | 5 | No | 6 | 3 | 1 | 6 | 11 | 0 | 0 |
| VNS+TAU | Homicide | 30.8 | M | 16 | 12 | No | 9 | 6 | 1 | 7 | 43 | 3 | 0 |
